# Chilblains in COVID-19 Infection

**DOI:** 10.7759/cureus.9245

**Published:** 2020-07-17

**Authors:** Vinuta Mohan, Robert Lind

**Affiliations:** 1 Internal Medicine, Department of Endocrinology, Saint Francis Hospital and Medical Center, Trenton, USA; 2 Diabetes and Endocrine Associates of Hunterdon, Hunterdon Medical Center, Flemington, USA

**Keywords:** chilblain, pediatrics, covid 19, skin disease/ dermatology

## Abstract

COVID-19 has quickly become a worldwide pandemic and is well-known for its pulmonary complications. Dermatologic manifestations such as chilblain-like lesions have been recently described, but it is unclear if they are truly secondary to the infection or not. Here we describe a young patient who developed chilblain-like eruptions on his toes, likely secondary to severe acute respiratory syndrome coronavirus 2 (SARS-CoV-2) infection. We discuss the literature that supports the hypothesis that these are in fact secondary to the infection, as well as provide insight into the pathology of these lesions.

## Introduction

Severe acute respiratory syndrome coronavirus 2 (SARS-CoV-2), which causes the infection COVID-19, originated in China in December 2019. It has become a worldwide pandemic unlike any other of our time. It is highly contagious and has spread quickly throughout the globe, causing overwhelming morbidity and mortality. Transmission of the infection occurs by inhalation of infected droplets or direct contact with soiled surfaces or fomites. The most common symptoms are fever, shortness of breath and cough. Headache myalgia, anosmia, asthenia and diarrhea are also often reported. However, many patients are asymptomatic, making it difficult to identify and isolate infected individuals. The disease seems to be milder in children as compared to adults, although infants and younger children tend to have a more severe illness than older children [1,2]. 

Cutaneous complications related to COVID-19 were not initially described. However, reports of new-onset chilblain-like lesions of upper and lower extremity digits emerged from Europe in the spring of 2020 [3]. Chilblains are small eruptions caused by the inflammation of tiny blood vessels after exposure to cold air. They usually appear on the hands and feet and are associated with pain and burning sensations.

Interestingly, many of these patients tested negative for SARS-CoV-2 infection. However, despite the negative test results, COVID-19 was still suspected because chilblains usually appear in the cold weather and these lesions occurred during the warmer spring weather. Furthermore, all patients lacked a previous history of chilblains, Raynaud syndrome, or collagen vascular disease. This occurrence was so strongly suspected to be secondary to COVID-19 it has been labelled as "COVID toes" in public media outlets [4]. Here we describe a young patient we suspect also had COVID-19 associated chilblain-like lesions.

## Case presentation

A 10-year-old male child presented to his medical provider with approximately one week of fever associated with documented temperatures 100-102 degrees Fahrenheit. His fever was associated with fatigue, generalized body aches, and dry cough. His symptoms started approximately three days after he and his parents had returned from vacation at an indoor water park facility. He had no significant past medical history other than seasonal allergies and was on no chronic medications. He had no remarkable surgical, social, or family medical history. He was presumptively diagnosed with a flu-like viral illness and instructed to rest and use over the counter pain relievers. With supportive care, his fever and symptoms gradually improved. 

**Figure 1:**
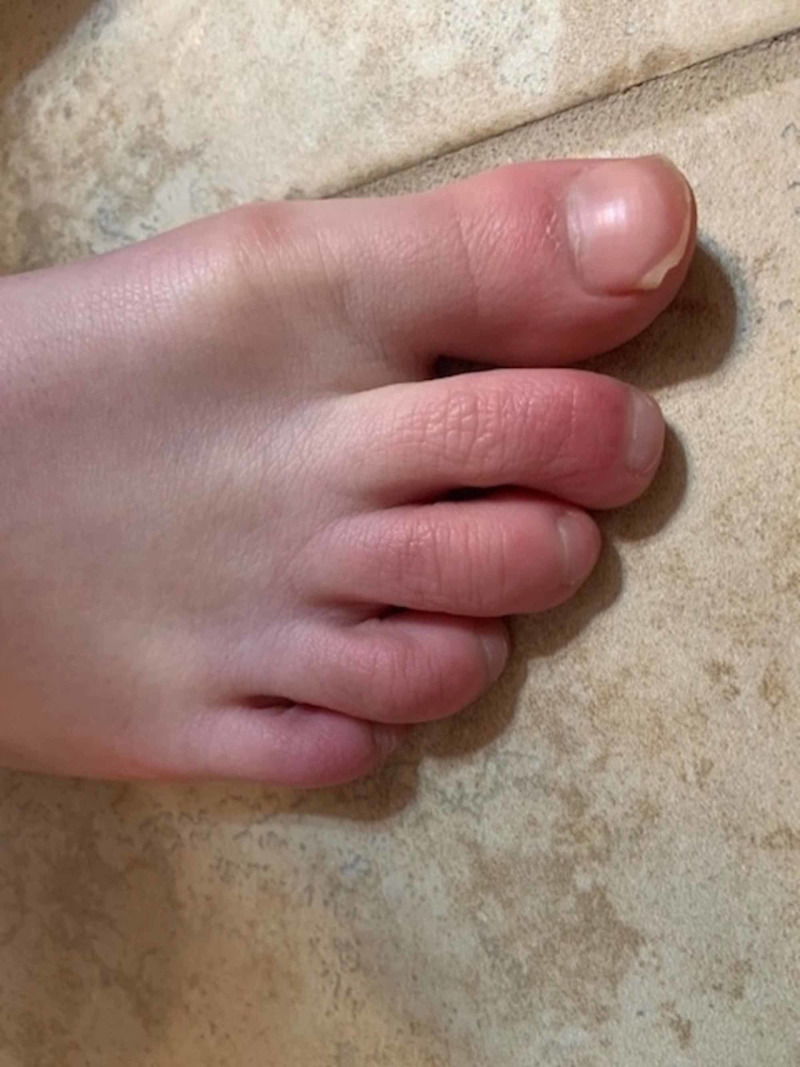
Chilblain-like lesions of the right foot

**Figure 2:**
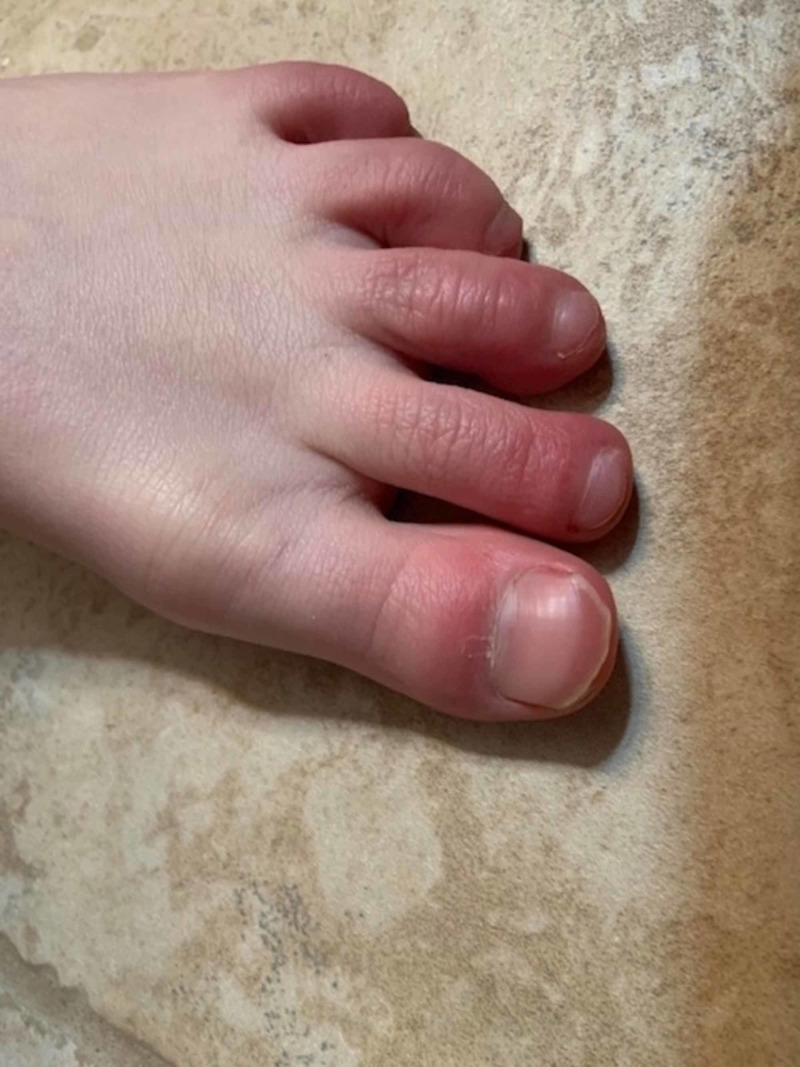
Chilblain-like lesions of the left foot

However, approximately two weeks after his initial symptoms started, the patient reported new onset of erythema, swelling, burning, and itching of the digits of both feet (Figure 1, Figure 2). His symptoms were worse at night and he had difficulty sleeping. He returned to his medical provider, was diagnosed with Raynaud syndrome, and warm socks were recommended. Unfortunately, his symptoms actually worsened with the suggested treatment and he again returned to the provider about two weeks later.

**Figure 3:**
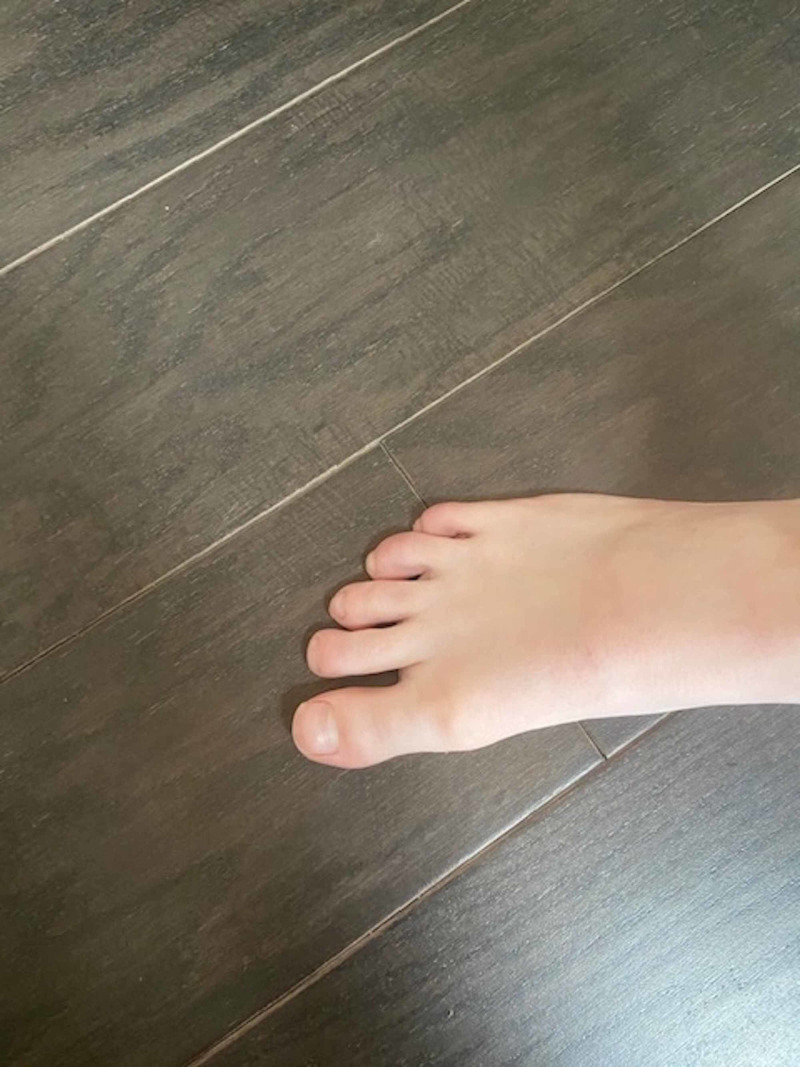
Follow-up of chilblain-like lesions of right foot

**Figure 4:**
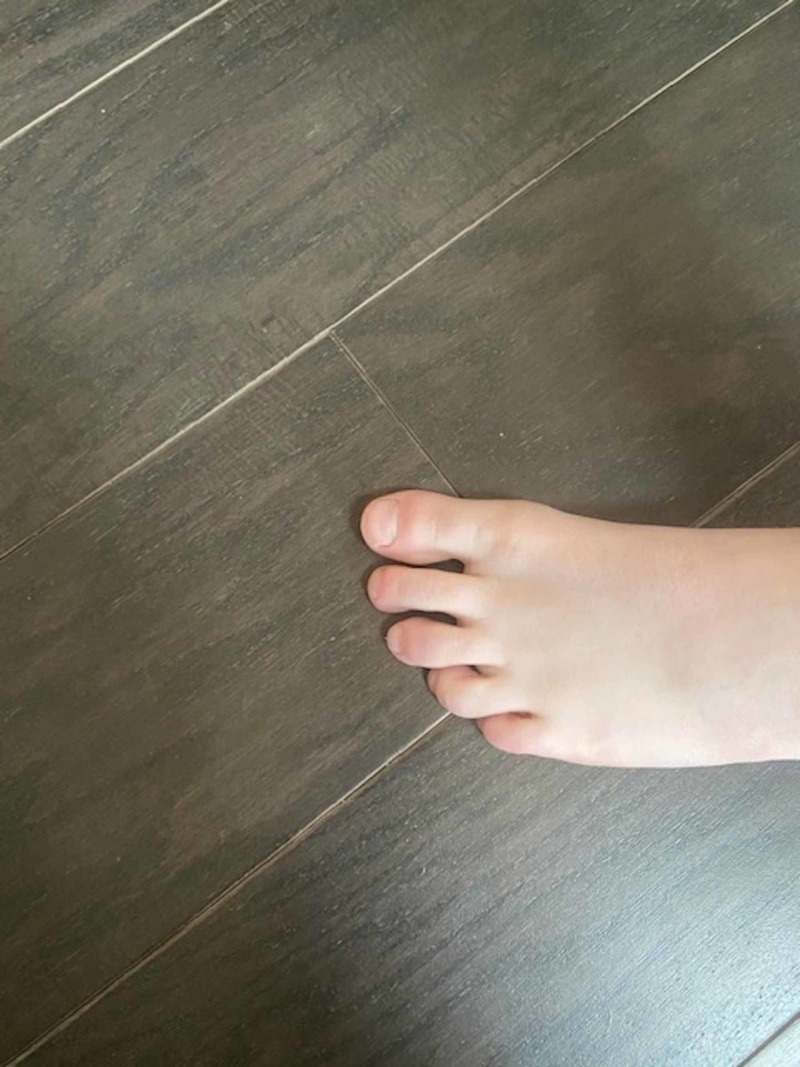
Follow-up of chilblain-like lesions of left foot

By this time, the COVID-19 pandemic was well established and cutaneous manifestations of the infection were reported. He underwent reverse transcription polymerase chain reaction (RT-PCR) SARS-CoV-2 and SARS-CoV-2 serology testing, both of which were negative. Despite the negative results, the diagnosis of COVID-19 was suspected and a topical glucocorticoid cream was prescribed. To date, the patient's lower extremity symptoms have persisted for approximately two months with significant improvement, but not full resolution (Figure 3, Figure 4). Both of his parents were asymptomatic throughout this time period and were never evaluated for SARS CoV-2 infection.

## Discussion

Soon after the COVID-19 pandemic started, multiple case series in Europe reported the new onset of cutaneous chilblain-like lesions, especially in the lower extremities, in children and young adults. Two different reports from Spain (one report described six patients, the other 22 patients) described patients who presented with erythematous, edematous papular skin lesions mostly on their toes and soles of their feet. The lesions were often associated with pruritis and pain. Most of the patients were children and young adults. The patients did not show any signs of Raynaud syndrome or ischemia and were generally in good health. Most patients did not present with classic COVID-19 symptoms but some did have mild fever or congestion prior to the onset of skin lesions. Access to SARS-CoV-2 RT-PCR testing was unavailable to most of these patients, but three patients did test positive in the smaller study and only one patient tested positive in the larger study [5,6].

Another report of the outbreak of chilblain-like lesions came from Italy involving 63 patients. The patients were young (median age 14 years old) and had mostly feet involvement. The most common presentation was erythematous-violaceous lesions associated with pain and itching. Many of the patients also had gastrointestinal symptoms, respiratory symptoms and/or fever. In most cases, the systemic symptoms preceded the cutaneous findings. SARS-CoV-2 testing was unavailable for many of the patients in this report as well. Only 17 patients were tested and two were positive for SARS-CoV-2 RT-PCR testing and two had positive serology [7].

A larger retrospective study from France looked at the patients with new-onset skin lesions during the COVID-19 pandemic, including 106 patients with chilblain-like lesions on hands and feet. The median age of the patients was 27 years old. Similar to the other reports, many of the patients had a fever or respiratory symptoms preceding the chilblains and the majority had no previous significant medical history. In this study, only 25 patients had RT-PCR SARS-CoV-2 testing performed and seven out of the 25 patients were positive [8].

In these reports, there was no recent exposure to cold temperatures, the risk factor for typical chilblains. As the outbreak of these lesions coincided with the COVID-19 spread, SARS-CoV-2 was hypothesized as being most likely responsible. Since these lesions were reported after the respective regions reached peak COVID-19 infection, chilblain-like lesions were likely a late manifestation of the disease. This theory was supported by the fact that many patients did report flu-like symptoms weeks before the occurrence of skin lesions, as our patient did. This may explain why testing at the time of the skin lesions was negative in affected patients; they were being tested at a time when the viral load and infectivity were low.

Some recent case reports have disputed the notion that the chilblain-like lesions were secondary to SARS-CoV-2 infection because the patients in these series tested negative for both RT-PCR and serology testing. The authors speculated the development of chilblains was due to decreased physical activity and being barefoot during quarantine [9,10]. However, the majority of patients did not have clinical symptoms consistent with COVID-19 prior to the onset of lesions as our patient did. Furthermore, our patient's lesions began before the quarantine period, so he was not barefoot or inactive for prolonged periods of time when his chilblains developed.

The exact pathology for chilblains secondary to COVID-19 infection is unknown but IFN-1 activation may play a role. Biopsies of these lesions have shown superficial and deep lymphoplasmacytic infiltrates similar to chilblains seen in chilblain lupus, a rare type of cutaneous lupus erythematosus (CLE). COVID-19 triggers the expression of type 1 interferon (IFN-1) genes as a part of a patient's anti-viral defense. IFN-1 genes are also linked to the development of systemic lupus erythematosus (SLE). So it is possible that young patients may exhibit a robust and rapid IFN-1 response to SARS-CoV-2 infection, which in turn inhibits viral replication and leads to a somewhat less severe course of the disease. 

However, this strong viral response may also likely induces small vessel changes after the initial infection, producing chilblain-like lesions. This differs from the ischemia and thrombosis often observed in infected older adult patients. The strong IFN-1 response in young patients may be advantageous, and the appearance of COVID-19 infection-induced chilblains may be associated with an indolent course and good prognosis [11]. But caution must be made before making any definite conclusions because the number of patients reported having these lesions remains low. 

## Conclusions

Hundreds of thousands have died and millions of cases have been reported worldwide in the COVID-19 pandemic. It is still unclear if the outbreak of chilblain-like lesions are directly caused by COVID-19 infection or not. Regardless, medical providers must have a heightened suspicion for SARS-CoV-2 infection and consider it in their patients' differential diagnoses as the list of atypical COVID-19 associated signs and symptoms continue to grow.
